# Safety and Efficacy of the PrePex Male Circumcision Device: Results From Pilot Implementation Studies in Mozambique, South Africa, and Zambia

**DOI:** 10.1097/QAI.0000000000000742

**Published:** 2016-05-24

**Authors:** Paul Feldblum, Neil Martinson, Bruce Bvulani, Noah Taruberekera, Mehebub Mahomed, Namwinga Chintu, Minja Milovanovic, Catherine Hart, Scott Billy, Edgar Necochea, Alick Samona, Miriam Mhazo, Debora Bossemeyer, Jaim Jou Lai, Limakatso Lebinai, Tigistu A. Ashengo, Lucinda Macaringue, Valentine Veena, Karin Hatzold

**Affiliations:** * Global Health Department, FHI 360, Durham, NC;; † Peri-natal HIV Research Unit, Faculty of Health Sciences, University of Witswatersrand, Johannesburg, South Africa;; § University Teaching Hospital, Lusaka, Zambia;; ‖ Population Services International, Washington, DC;; ¶ Jhpiego, Maputo Moçambique;; # Society for Family Health, Lusaka, Zambia;; ** Population Services International Lusaka, Zambia;; ‡‡ Society for Family Health, Johannesburg, South Africa;; §§ Jhpiego, Baltimore, MD;; ‖‖ Population Services International, Maputo, Mozambique;; ¶¶ FHI 360, Nairobi, Kenya; and; ## Population Services International, Harare, Zimbabwe.

**Keywords:** PrePex, male circumcision, safety, Mozambique, Zambia, South Africa

## Abstract

**Background:**

Fourteen countries in East and Southern Africa have engaged in national programs to accelerate the provision of voluntary medical male circumcision (VMMC) since 2007. Devices have the potential to accelerate VMMC programs by making the procedure easier, quicker, more efficient, and widely accessible.

**Methods:**

Pilot Implementation studies were conducted in Mozambique, South Africa, and Zambia. The primary objective of the studies was to assess the safety of PrePex device procedures when conducted by nurses and clinical officers in adults and adolescent males (13–17 years, South Africa only) with the following end points: number and grade of adverse events (AEs); pain-related AEs measured using visual analog score; device displacements/self-removals; time to complete wound healing; and procedure times for device placement and removal.

**Results:**

A total of 1401 participants (1318 adult and 83 adolescent males) were circumcised using the PrePex device across the 3 studies. Rates of moderate/severe AEs were low (1.0%; 2.0%; and 2.8%) in the studies in Mozambique, Zambia, and South Africa, respectively. Eight early self-removals of 1401 (0.6%) were observed, all required corrective surgery. High rates of moderate/severe pain-related AEs were recorded especially at device removal in South Africa (34.9%) and Mozambique (59.5%). Ninety percent of participants were healed at day 56 postplacement.

**Discussion:**

The study results from the 3 countries suggest that the implementation of the PrePex device using nonphysician health care workers is both safe and feasible, but better pain control at device removal needs to be put in place to increase the comfort of VMMC clients using the PrePex device.

## BACKGROUND

Three randomized controlled trials have demonstrated that male circumcision reduces the risk of heterosexually acquired HIV infection in men by 60%.^1–3^ Fourteen countries in East and Southern Africa have engaged in national programs to accelerate the provision of voluntary medical male circumcision (VMMC) since 2007. VMMC is a 1-time intervention resulting in lifelong protection from HIV infection in men. However, despite the compelling evidence, the progress of scaling up VMMC across the 14 countries has been variable. It is estimated that by December 2013, 6 million male circumcisions had been conducted against a target of 20.3 million.^4,5^

Male circumcision devices have the potential to accelerate VMMC programs by making the procedure easier, quicker, more efficient, and widely accessible.^6–8^

One promising device is PrePex, a nonsurgical circumcision device, developed by Circ MedTech. It is an elastic collar compression device, which works through slow compression of the foreskin between an outer elastic ring and inner hard surface between glans and foreskin, which occludes the circulation and produces tissue devitalization and necrosis.^9^ The device is left in place for 7 days during which the compressed foreskin becomes necrotic and then is removed by cutting with scissors; the device can be applied and the foreskin and the device can be removed without injected local anesthetic.^9^

The World Health Organization Technical Advisory Group (WHO TAG) reviewed 8 published and unpublished studies of the PrePex device in January, 2013. Based on the available data from Rwanda, Zimbabwe, and Uganda, the TAG provided conditional prequalification for use of the PrePex device in men aged 18 or older, but recommended that skilled surgical backup be available to manage severe complications.^7,10–13^

After the recommendations by WHO and the TAG outlined in the *Framework for Clinical Evaluation of Devices for Male Circumcision*,^14,15^ pilot implementation studies using the PrePex device were conducted in all VMMC focus countries except Ethiopia, Namibia, and Swaziland. The aim was to establish the feasibility and the acceptability of the new device for the program, providers, clients, their families, and partners. The primary objective of the studies was to assess the safety of PrePex MC procedures conducted by nurses and clinical officers during routine service delivery in adult males. We present results from 3 pilot implementation studies with a total sample size of 1401.

## METHODS

### Study Design

In all 3 countries, a single arm, open label, prospective, cohort study was conducted. In Mozambique, a total of 504 males aged 18 to 49 were recruited at one fixed study site, José Macamo General Hospital in Maputo. In South Africa, 315 adult males (18–45) were recruited at VMMC sites at Witbank Hospital, Tsakane Clinic, and Zuzimpilo Clinic in Johannesburg. An additional 83 adolescent participants (13–17 years) were recruited at Tsakane and Zuzimpilo. In Zambia, the PrePex study was conducted among 499 adult males (18–49 years) at 2 fixed VMMC clinics in the capital city of Lusaka, which were managed by the Society for Family Health (SFH).

PrePex circumcisions were conducted at existing fixed VMMC sites where conventional surgical VMMC services were also provided to facilitate immediate access to care in case of a serious adverse event (SAE) requiring surgical correction. Written informed consent was sought from all participants in the different studies and VMMC staff at all sites were trained in the PrePex procedure per standard protocol provided by the manufacturer. PrePex circumcisions were conducted by nurses, clinical associates, and doctors in South Africa, by nurses in Mozambique, and by nurses and clinical officers in Zambia.

Follow-up of study participants differed across the 3 country studies. All review days were referenced to postplacement with the day of placement set at day 0. Device removals were scheduled at day 7 at all sites. In Mozambique, participants were asked to return for additional clinical visits at days 28 and 49, thereafter participants were asked to return to the clinic for review until complete wound healing. Interviewers also made follow-up calls to clients on days 14 and 21. In South Africa, study participants were followed up after device removal weekly at the clinic until complete wound healing was observed. In Zambia, the first 50 participants were followed intensely at 5 follow-up visits (days 9, 14, 21, 35, and 42), and the remaining men were followed at days 7 and 42 per routine service delivery. Men were encouraged to return to the clinic at any time if they faced challenges or had concerns in all studies.

### Outcome Measures

Outcome measures included:

- Frequency and percentage of adverse events (AEs). Per WHO recommendations,^15^ we measured the rate of moderate and severe AEs. All AEs were recorded and classified as mild, moderate, and severe according to the PSI/COSECSA AEs Action Guide for VMMC as modified for other PrePex pilot implementation studies.^16^ AEs included those due to pain during the PrePex process; measured using a visual analog score with a range of 0–10, where 0 corresponds to “no pain at all” and 10 to “worst pain imaginable.” Clients were shown pictograms for 6 different rating levels. The pain assessments were made at specified time points throughout the study.
- Frequency and percentage of device displacements and self-removals were included under AEs and device malfunctions. Difficult placements were recorded separately from AEs.
- Proportion of screening failures and reasons for participant exclusion.
- Time taken for placement and removal of the device. In Mozambique and Zambia, time of device placement was recorded from application of the anesthetic cream to cutting of the verification thread, whereas in South Africa, time of device placement was measured from the start of cleaning of the genitalia, to the client leaving the operating table with the device applied.
- Frequency and proportion of men healed at different time points. A participant was classified as healed if the wound was completely epithelialized. Whether complete wound healing had occurred was assessed through clinical examination on agreed review dates or through telecommunication. If the clients had not healed on the specific review date, they were asked to return for review weekly until complete wound healing was achieved.

### Recruitment and Eligibility

All 3 studies followed similar study and data collection procedures. Males were recruited from the surrounding areas of VMMC PrePex study sites. All clients received information during individual and group sessions on VMMC and were offered 2 options, either standard surgical procedure or PrePex device male circumcision. Those choosing surgery received the conventional surgical standard of care (nonresearch). Individual consent for inclusion in the PrePex study was obtained from those interested in participating in the study, parent consent and participant assent were obtained from adolescent participants in the South Africa study. Clients were offered HIV testing, and all clients were then screened for PrePex eligibility.

### Inclusion and Exclusion Criteria

The following inclusion and exclusion criteria were used in all 3 studies.

#### Inclusion Criteria

Men aged 18–49 (Mozambique and Zambia) and 13–45 years (South Africa), uncircumcised, consents/assents to the PrePex male circumcision procedure, HIV-negative—in good general health and clinically free of sexually transmitted infections (STI)—provides contact information, agrees to active follow-up, providing location information and a cell phone number.

#### Exclusion Criteria

Penis did not fit any of the 5 PrePex sizes; medical contraindication, cognitive, or psychiatric impairment as determined by staff, genital anatomic abnormalities or/and active genital disease/infections, evidence of partial circumcision, or scarification.

### Ethics Approvals

The study protocol in Mozambique was approved by the Mozambican National Bioethics Committee (Reference 73/CNBS/13 dated April 8, 2013), by the Johns Hopkins University IRB (Reference JHSPH IRB-FC IRB No: 00004637 dated January 2, 2013) and by the CDC Center for Global Health (May 28, 2013). The study received administrative approval from the Ministry of Health (MOH) (Reference 591/GMS/002/2012). The study in South Africa was approved by the University of the Witwatersrand's Human Research Ethics Committee and South African provincial level research committees. The study in Zambia was reviewed and approved by the IRBs of FHI 360 and the University of Zambia Biomedical Research Ethics Committee (UNZABREC), as well as by the Zambian MOH and the Zambian Medicines Regulatory Authority (ZAMRA), which approved importation of the devices into Zambia.

## RESULTS

### Study Populations, Eligibility

Baseline data of clients, sample sizes, study sites, timing of the studies are included in Table1.

**TABLE 1.**
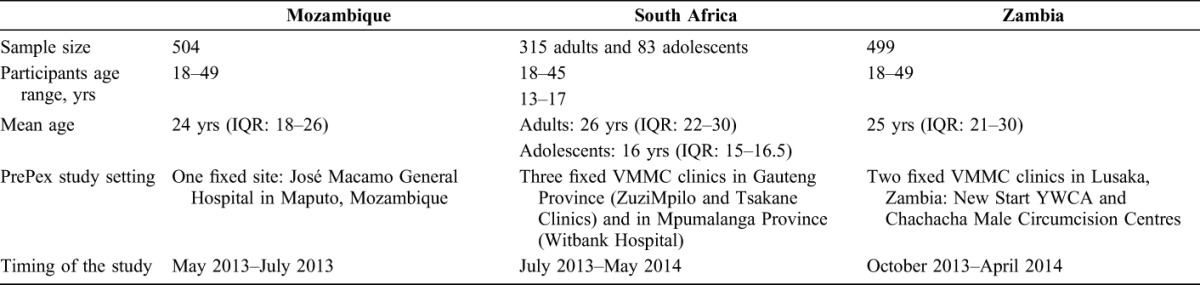
Baseline Data of Clients, Sample Sizes, Study Sites, Timing of the Study

#### Mozambique

608 clients attending the VMMC clinic at José Macamo General Hospital in Maputo were consented, enrolled in the study, interviewed, and screened for eligibility. Of these, 104 (17.1%) were determined to be ineligible and 504 (82.9%) had the PrePex device placed. The 104 clients screened out included 65 clients with HIV seropositive status, 12 clients with phimosis and narrow foreskin, 3 with symptomatic STIs, 1 with mental impairment, 17 participants were ineligible because of other social reasons challenging follow-up, and an additional 6 participants had to be excluded after reverification of their age, as they were below 18 years of age.

#### South Africa

A total of 454 males, 339 (74.7%) adults (18–45 years), and 94 (25.3%) adolescents (13–17 years) were assessed for eligibility to receive the PrePex device across the 3 VMMC/PrePex study sites. Of those assessed, 21 (4.6%) refused PrePex circumcision during the consenting process; 433 (95.4%) consented and then screened, of whom 35 (7.7%) failed the screening process due to medical or social reasons. Of the 35 ineligible clients, 24 (68.6%) were adults and 11 (31.4%) were adolescents (13–17 years).

#### Zambia

A total of 546 men who presented at the 2 Lusaka SFH VMMC clinics agreed to undergo circumcision by PrePex. Of these men, 500 (91.6%) received PrePex male circumcision. There were 18 (3.3%) exclusions due to phimosis, hypospadias, narrow foreskin, or other anatomic condition; 9 (1.7%) with symptomatic STI; 17 (3.1%) with other medical contraindications; one client (0.2%) withdrew consent; and 1 (0.2%) did not receive PrePex for an unspecified reason. One HIV-infected man inadvertently received PrePex, and he was excluded from the analysis, although he was followed for healing and safety in the same fashion as other participants.

### Safety

#### Adverse Events

Table 2 summarizes moderate and severe AEs that occurred during the studies. A total of 26 (1.9%) participants experienced one or more moderate or severe AEs. Of the 26 participants who experienced at least one AE, 20 experienced one AE (either severe or moderate), three men had two severe AEs, one man had one severe and one moderate AE and two men reported three AEs, one severe and two moderate each. The moderate and severe AE rates for the 3 country studies were reported as follows: Mozambique 5/504 (1.0%), South Africa 11/398 (2.8%), and Zambia 10/499 (2.0%). There was no significant difference in the AE rate among the countries.

**TABLE 2.**
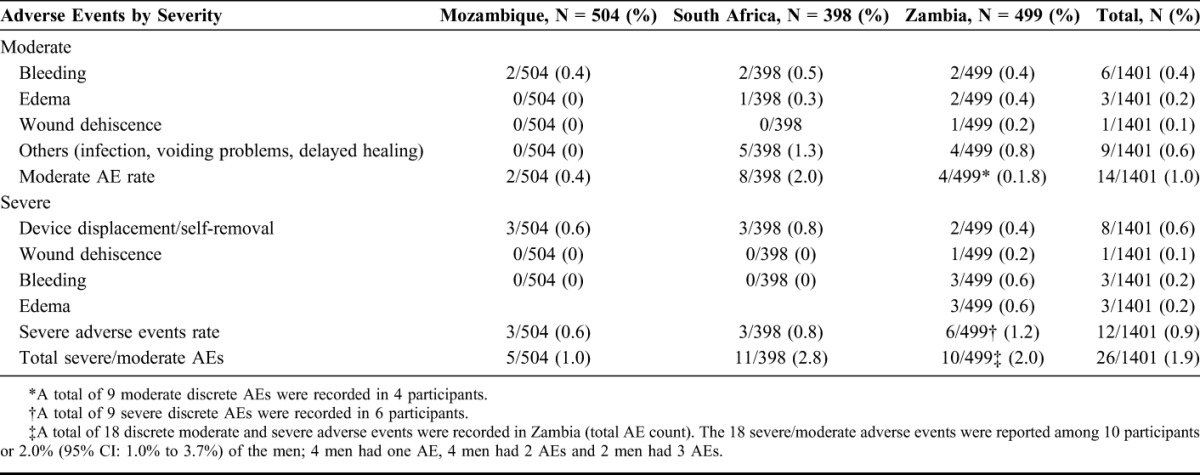
Number of Distinct Moderate and Severe Adverse Events Among All Participants With Device Placement, by Country (N = 1401) and Moderate and Severe Adverse Events Rate (%) by Country

A total of 34 distinct moderate (19) and severe (15) adverse events were observed across the 3 studies. There were 8 device displacements and/or self-removal, requiring corrective surgery, all were classified as severe adverse events. All resolved with subsequent uneventful healing. The other severe AEs included 3 distinct cases of bleeding (2 of which required suturing), 3 cases of edema, and one case of wound dehiscence. There were 19 distinct cases of moderate AEs, which included bleeding, edema, wound dehiscence, infection, voiding problems, and delayed wound healing. All reported AEs resolved with appropriate clinical intervention and without permanent sequelae.

#### Pain-Related AEs

Table 3 summarizes pain-related moderate and severe AEs. A total of 182/1401 (13%) moderate and 239/1401 (17%) severe pain-related AEs were reported, respectively. A higher number of pain-related AEs were reported from Mozambique and South Africa. Mozambique reported 300/504 (59.5%) and South Africa reported 139/398 (34.9%) moderate or severe pain-related AEs, whereas Zambia reported 2/499 (0.4%) moderate pain-related AEs, most of which occurred at device removal.

**TABLE 3.**
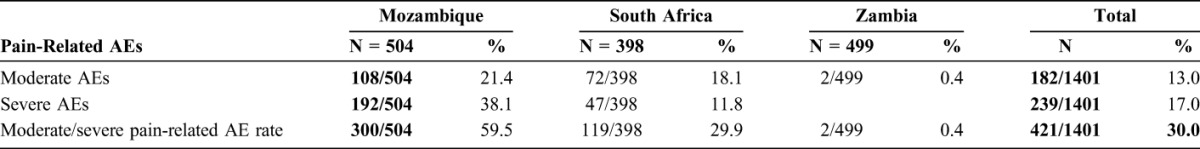
Pain-Related Moderate and Severe Adverse Events Among all Participants With Device Placement by Country and Moderate and Severe Pain-Related Adverse Events Rate (%) by Country

### Placement and Removal Procedure

The mean time for the placement procedure varied between 2.7 and 3.1 minutes in Mozambique, 7 minutes in South Africa, and 2.4 minutes in Zambia. The mean time for removal of the device and necrotized foreskin varied between 1.9 and 2.7 minutes in Mozambique, 2.4 minutes in South Africa, and 4.7 minutes in Zambia.

### Time to Complete Wound Healing

**FIGURE 1.**
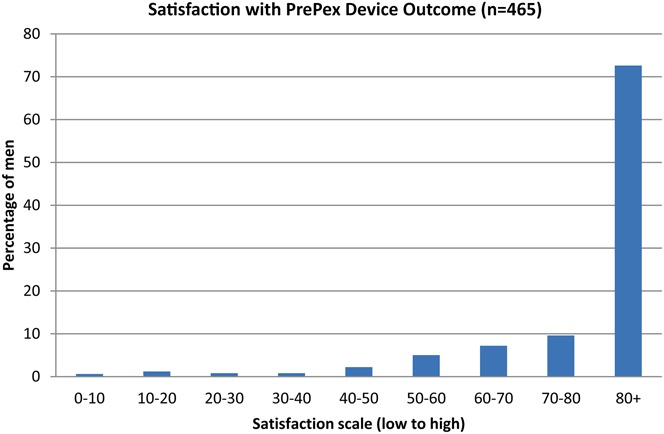
Cumulative proportion of adult participants with complete wound healing at 42, 49, and 56 days after device application.

Among adult study participants in South Africa and Zambia, respectively, 43/315 (13.5%) and 277/499 (55.5%) men were observed as completely healed at day 42 (Fig. 1). By day 49, 422/501 (84.2%), 122/315 (38.7%), and 438/499 (87.8%) of participants from Mozambique, South Africa, and Zambia, respectively, were reported to be completely healed. By day 56, 445/501 (88.8%) and 305/315 (96.7%) of the adult participants from Mozambique and South Africa, respectively, had completely healed.

**FIGURE 2.**
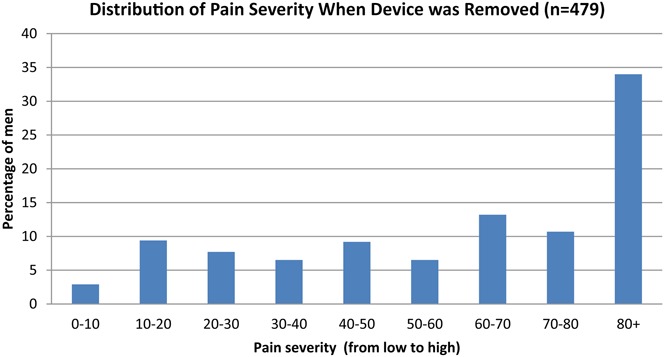
Cumulative proportion of adult and adolescent participants with complete wound healing at 42, 49, and 56 days post device application, South Africa.

Figure 2 shows that there was a significant difference in time to healing between adults and adolescents in the South African study, with adolescents healing faster than adults (*P* < 0.001).

Loss to follow-up of study participants, who should have presented at the day 42 review visit but were not seen, was 28/315 (9%) and 25/499 (5%) in South Africa and Zambia, respectively. In Mozambique, loss to follow-up at day 49 was 23/501 (4.6%).

## DISCUSSION

The moderate/severe AE rates from the 3 different studies in Mozambique (1.0%), South Africa (2.6%), and Zambia (2.0%) are close to the AE rate reported in other PrePex safety studies conducted in Rwanda,^9,17–19^ Uganda,^20,21^ and Zimbabwe,^11–13^ whereas overall moderate/severe AE rate in a recent study in Kenya^22^ was slightly higher (5.9%) including more device displacements/self-removals than the 3 studies here described (0.4% in Mozambique, 0.6% in South Africa, and 0.8% in Zambia).

Although the rate of displacements and self-removals was low (8/1401, 0.6%), they are not only severe but are defined as serious adverse events, necessitating surgical circumcision by sleeve or dorsal slit method. Programs must ensure that surgical circumcision backup is readily available to handle such cases. Special attention must be placed on appropriate counseling and adequate information about the risk related to sexual intercourse with device in situ and self-tampering with the device.

The overall moderate/severe AE rate in the 3 PrePex studies described here is also comparable with those reported for the surgical male circumcision procedure either with the forceps guided method^1,2,23^ or the dorsal slit method^3,24^ and is also similar to the AE rate in adults using the ShangRing.^25,26^

Most of 1401 PrePex procedures were conducted by midlevel providers (nurses, clinical officers, and clinical associates). This could be a potential advantage over male circumcision surgery, especially in VMMC priority countries where task shifting of the male circumcision procedure is not yet policy, as it could reduce the costs and reduce the human resources capacity challenges that VMMC programs in these countries are facing.

There were variations in placement and removal times over the different studies, which are explained by different timing protocols and by the different levels of experience by the providers involved. The mean total time (placement preparation and procedure time and removal preparation and procedure time) in each of the 3 studies was lower than the procedure time for conventional surgery (19.2 minutes).^10^ This represents another advantage over the surgical procedure in terms of efficiency, which could result in higher client turnover and outputs, thus reduced per VMMC unit costs.

Owing to the fact that wound healing after PrePex procedure is by secondary intention, the time required for complete healing across the 3 studies was at least about 1 week longer than that reported after surgical VMMC, for which more than 90% of men are judged healed by day 42.^27^ In these studies, 84.2%, 38.7%, and 87.8% of adult participants had complete wound healing on day 49 in Mozambique, South Africa, and Zambia, respectively. In previous studies conducted in Rwanda and Zimbabwe, mean time to complete healing was up to 2 weeks longer than with surgery, and the mean healing time after placement recorded over 5 PrePex studies was 42.3 days, which is comparable with the healing time that was recorded in the 3 studies presented here.^10^ Time to complete wound healing was significantly shorter in adolescents than in adults in the study from South Africa. Similar findings were observed in the PrePex adolescents study in Zimbabwe where mean time to complete wound healing was 31.9 days (SD = 5.5) in adolescents (13–17 years) as compared with 42.3 days (SD = 7.8) in adults.^26^ Longer healing time could potentially result in higher risk of HIV acquisition in circumcised men during the healing period and represents also a higher risk of HIV transmission to female partners of recently circumcised HIV positive men.

The PrePex device was safe and effective for circumcision in adult men and a small cohort of adolescent males in South Africa. The studies provide better understanding of some of the reported advantages of the method, including ease of task shifting to nonphysician cadres of providers; increased efficiency due to reduced procedure time; and lower risk of bleeding and infection as compared with surgery. Nevertheless, the studies reported high rates of pain-related AEs, especially at device removal, which needs to be addressed. Pain-related AEs were much more frequently reported in Mozambique and in South Africa as compared with Zambia. This might be explained by the use of different pain-related AE classifications used across the studies and variability of interpretation by providers. Use of topical anesthetic cream in combination with oral analgesics at device removal should be evaluated. Pain related to the device procedure could lead to reduced acceptability of the device among potential clients and needs to be addressed urgently. It is important to note that backup surgery by an experienced provider proficient in the dorsal slit or sleeve method needs to be available because of the risk of device displacement and self-removal by the client. Additional research to better understand barriers and motivators for uptake of PrePex will inform demand creation and communications strategies to promote device VMMC. Results from acceptability studies that were nested within the pilot implementation studies for which data are presented here are described separately to this manuscript.^27^ The PrePex device VMMC performed well in all 3 country studies and is safe when used alongside routine VMMC surgical service delivery. The device has the potential to facilitate widespread scale-up of safe VMMC in sub-Saharan Africa.

We note the following study limitations:

Interpretation of complete healing and pain scores was subjective and could vary between MC providers and clinics as well as across countries.^28^ This explains the high variability for the pain-related AEs reported from the 3 countries. Because of the different methodologies in measuring timing of the procedures by country, it is impossible to compare timing across the 3 countries. The most important weakness of the study was the incompleteness of postremoval follow-up of study participants across all studies. Follow-up of each participant was intended until complete healing could be confirmed, but a high percentage of the men were either lost to follow-up after device removal or exited the study without certification of complete healing.

## References

[1] Auvert B Taljaard D Lagarde E Randomized, controlled intervention trial of male circumcision for reduction of HIV infection risk: the ANRS 1265 Trial. PLoS Med. 2005;2:e298. 16231970 10.1371/journal.pmed.0020298 PMC1262556

[2] Bailey RC Moses S Parker CB Male circumcision for HIV prevention in young men in Kisumu, Kenya: a randomised controlled trial. Lancet. 2007;369:643–656. 17321310 10.1016/S0140-6736(07)60312-2

[3] Gray RH Kigozi G Serwadda D Male circumcision for HIV prevention in men in Rakai, Uganda: a randomised trial. Lancet. 2007;369:657–666. 17321311 10.1016/S0140-6736(07)60313-4

[4] Sgaier SK Reed JB Thomas A Achieving the HIV prevention impact of voluntary medical male circumcision: lessons and challenges for managing programs. PLoS Med. 2014;11:e1001641. 24800840 10.1371/journal.pmed.1001641 PMC4011573

[5] Njeuhmeli E Hatzold K Gold E Lessons learned from scale-up of voluntary medical male circumcision focusing on adolescents: benefits, challenges, and potential opportunities for linkages with adolescent HIV, sexual, and reproductive health services. J Acquir Immune Defic Syndr. 2014;66:S193–S199. 24918595 10.1097/QAI.0000000000000179

[6] McIntyre JA Can devices for adult male circumcision help bridge the implementation gap for HIV prevention services? J Acquir Immune Defic Syndr. 2011;58:506–508. 21963938 10.1097/QAI.0b013e318237af5d

[7] WHO. Guideline on the Use of Devices for Adult Male Circumcision for HIV Prevention. 2013. 24624479

[8] Njeuhmeli E Kripke K Hatzold K Cost Analysis of Integrating the PrePex Medical Device into a Voluntary Medical Male Circumcision Program in Zimbabwe. PLoS ONE. 2014;9:e82533. 10.1371/journal.pone.0082533. 24801515 PMC4011574

[9] Mutabazi V Kaplan SA Rwamasirabo E HIV prevention: male circumcision comparison between a nonsurgical device to a surgical technique in resource-limited settings: a prospective, randomized, nonmasked trial. J Acquir Immune Defic Syndr. 2012;61:49–55. 22739133 10.1097/QAI.0b013e3182631d69

[10] WHO. WHO Technical Advisory Group on Innovations in Male Circumcision; Evaluation of Two Adult Devices. Geneva, Switzerland: WHO; 2013.

[11] Tshimanga M Gwinji G Mugurungi O A Randomized Controlled Trial Comparing the PrePex Device to Forceps Guided Surgical Circumcision for Rapid Scale-Up of Male Circumcision in Zimbabwe. Phase II: Randomized Trial Report*.* Harare, Zimbabwe: Ministry of Health and Child Welfare; 2013.

[12] Tshimanga M Gwinji G Mugurungi O Evaluation of Safety and Efficacy of PrePex Device for Male Circumcision inZimbabwe. Phase I: Device Safety Trial Report*.* Harare, Zimbabwe: Ministry of Health and Child Welfare; 2013.

[13] Tshimanga M Gwinji G Mugurungi O Evaluation of Safety and Efficacy of PrePex Device for Male Circumcision inZimbabwe. Phase 3: Cohort Field Study on Safety and Efficacy of the PrePex Circumcision Device When Performed by Nurses*.* Harare, Zimbabwe: Ministry of Health and Child Welfare; 2013.

[14] WHO. WHO Prequalification of Male Circumcision Devices Public Report. Product: PrePex. Geneva, Switzerland: WHO; 2013.

[15] WHO/UNAIDS. Framework for Clinical Evaluation of Devices for Adult Male Circumcision. Geneva, Switzerland: WHO/UNAIDS; 2010.

[16] PSI/COSECSA. Adverse Events Action Guide for Voluntary Medical Male Circumcision. Harare, Zimbabwe: PSI/COSECSA; 2013.

[17] Mutabazi V Bitega JP Ngeruka LM Non-surgical adult male circumcision using the PrePex device: task-shifting from physicians to nurses. Afr J Reprod Health. 2014;18:61–70. 24796170

[18] Mutabazi V Kaplan SA Rwamasirabo E One-arm, open-label, prospective, cohort field study to assess the safety and efficacy of the PrePex device for scale-up of nonsurgical circumcision when performed by nurses in resource-limited settings for HIV prevention. J Acquir Immune Defic Syndr. 2013;63:315–322. 23466648 10.1097/QAI.0b013e31828e6412

[19] Bitega JP Ngeruka ML Hategekimana T Safety and efficacy of the PrePex device for rapid scale-up of male circumcision for HIV prevention in resource-limited settings. J Acquir Immune Defic Syndr. 2011;58:e127–134. 21909032 10.1097/QAI.0b013e3182354e65

[20] Galukande M Duffy K Bitega JP Adverse events profile of PrePex a non-surgical device for adult male circumcision in a Ugandan urban setting. PLoS One. 2014;9:e86631. 24489754 10.1371/journal.pone.0086631 PMC3904949

[21] Kigozi G Musoke R Watya S The safety and acceptance of the PrePex device for non-surgical adult male circumcision in Rakai, Uganda. A non-randomized observational study. PLoS One. 2014;9:e100008. 25144194 10.1371/journal.pone.0100008 PMC4140666

[22] Feldblum PJ Odoyo-June E Obiero W Safety, effectiveness and acceptability of the PrePex device for adult male circumcision in Kenya. PLoS One. 2014;9:e95357. 24788898 10.1371/journal.pone.0095357 PMC4006910

[23] Krieger JN Bailey RC Opeya JC Adult male circumcision outcomes: experience in a developing country setting. Urol Int. 2007;78:235–240. 17406133 10.1159/000099344

[24] Buwembo DR Musoke R Kigozi G Evaluation of the safety and efficiency of the dorsal slit and sleeve methods of male circumcision provided by physicians and clinical officers in Rakai, Uganda. BJU Int. 2012;109:104–108. 21627752 10.1111/j.1464-410X.2011.10259.x PMC4326085

[25] Kigozi G Musoke R Watya S The acceptability and safety of the Shang Ring for adult male circumcision in Rakai, Uganda. J Acquir Immune Defic Syndr. 2013;63:617–621. 23614991 10.1097/QAI.0b013e3182968dda PMC3805675

[26] Tshimanga M Hatzold K Mugurungi O Safety profile of PrePex male circumcision device and client satisfaction with adolescent males aged 13-17 years in Zimbabwe. J Acquir Immune Defic Syndr. 2016;72(suppl1):S36–S42. 27331588 10.1097/QAI.0000000000000799 PMC4936434

[27] Cummings B Necochea E Ferreira T Acceptability and satisfaction associated with the introduction of the PrePex circumcision device in Maputo, Mozambique. J Acquir Immune Defic Syndr. 2016;72(suppl1):S56–S62. 27331592 10.1097/QAI.0000000000000764 PMC4936425

[28] Sokal DC Li PS Zulu R Randomized controlled trial of the shang ring versus conventional surgical techniques for adult male circumcision: safety and acceptability. J Acquir Immune Defic Syndr. 2014;65:447–455. 24583615 10.1097/QAI.0000000000000061

